# Do Quiescence and Wasp Venom-Induced Lethargy Share Common Neuronal Mechanisms in Cockroaches?

**DOI:** 10.1371/journal.pone.0168032

**Published:** 2017-01-03

**Authors:** Stav Emanuel, Frederic Libersat

**Affiliations:** Department of Life Sciences, Ben-Gurion University of the Negev, Beer-Sheva, Israel; Universitat Regensburg, GERMANY

## Abstract

The escape behavior of a cockroach may not occur when it is either in a quiescent state or after being stung by the jewel wasp (*Ampulex compressa*). In the present paper, we show that quiescence is an innate lethargic state during which the cockroach is less responsive to external stimuli. The neuronal mechanism of such a state is poorly understood. In contrast to quiescence, the venom-induced lethargic state is not an innate state in cockroaches. The Jewel Wasp disables the escape behavior of cockroaches by injecting its venom directly in the head ganglia, inside a neuropile called the central complex a ‘higher center’ known to regulate motor behaviors. In this paper we show that the coxal slow motoneuron ongoing activity, known to be involved in posture, is reduced in quiescent animals, as compared to awake animals, and it is further reduced in stung animals. Moreover, the regular tonic firing of the slow motoneuron present in both awake and quiescent cockroaches is lost in stung cockroaches. Injection of procaine to prevent neuronal activity into the central complex to mimic the wasp venom injection produces a similar effect on the activity of the slow motoneuron. In conclusion, we speculate that the neuronal modulation during the quiescence and venom-induced lethargic states may occur in the central complex and that both states could share a common neuronal mechanism.

## Introduction

The Jewel Wasp uses live cockroaches, *Periplaneta americana*, as a food supply for its developing larva. Instead of using its venom to paralyze the cockroach and then facing the challenge of carrying the larger cockroach to the pre-selected burrow, the wasp manipulates the cockroach into behaving as a docile and compliant host for the upcoming handling by the wasp and feeding by the larva [1]. To accomplish this behavioral manipulation, the wasp injects its venom cocktail inside the head capsule, directly into both the sub-esophageal ganglion and the supra-esophageal ganglion (SupEG or “brain”) [2]. The sting to the SupEG is accurate and aimed to a neuropil termed the central complex (CX), a multisensory “higher center” known to regulate locomotion [3,4]. As a result, the cockroach enters a long-lasting lethargic state, which is characterized by a drastic decrease in its ability to initiate and maintain locomotion [5]. In addition, the cockroach displays a reduced responsiveness to stimuli. Specifically, stimuli which reliably evoke an escape response in an awake cockroach will, at best, induce a brief startle response in a stung cockroach (S1 File). This unresponsiveness to stimuli allows the wasp to further handle the cockroach, lead it to a burrow, and lay an egg on the cockroach's cuticle.

Interestingly, cockroaches display an innate quiescent state that is correlated to daytime and that behaviorally resembles the venom-induced lethargic state [6]. In this state, a cockroach exhibits diminished mobility and assumes a flaccid posture [6]. The neuronal mechanism of this quiescent state was first investigated by Watson and Ritzmann (1994) [6]. This quiescent state is inducible with a specific stimulus. When both of the cockroach's antennae touch the walls of an enclosed space, the cockroach enters a quiescent state. Quiescent- and venom-induced lethargic states bear some similarities in that the sensory information carried by abdominal giant interneurons is unaffected in both states [6,7]. Such abdominal giant interneurons (GIs), which span the length of the cockroach ventral nerve cord all the way to the brain, excite a group of thoracic interneurons that in turn excite leg motor neurons [8]. Among such leg motoneurons, the fast and slow coxal muscle depressor motoneurons (Df and Ds respectively) have been extensively studied in the context of both escape and locomotion [9–11]. Moreover, we have previously shown that cockroaches in a quiescent state resemble stung cockroaches in that, for both, the fast motoneuron of the leg depressor muscle involved in escape is never recruited [12]. Likewise, in both states, a wind stimulus evokes a short burst of the slow postural motoneuron or no motor response. Hence, the absence of motor responses characteristic of escape behavior and generated by the thoracic ganglia in both states must be controlled via descending input from the head ganglia [6,12].

The CX is known to receive multiple sensory inputs, including visual [13] and mechanical information from one or both antennae of insects, including cockroaches [14], bees [15] and locust [16]. It also appears to be involved in the ongoing regulation of locomotion [3,17–23]. It was shown recently that clock pacemaker neurons involved in regulating the rest-cycle in drosophila project to the CX, thereby regulating locomotor activity [24]. Hence, the CX could be the last station that controls multiple downstream circuits to orchestrate behavioral rhythms. Because the jewel wasp injects its venom cocktail into and around the CX, we hypothesize that it may have evolved the chemical arsenal to manipulate rest-wake homeostasis in its cockroach prey. In the present study, we first further characterize the cockroach quiescence state using behavioral and electrophysiological approaches. We then examine the neuronal correlates of the innate quiescence state and compare these with those of the venom-induced lethargic state to test whether the quiescent and stung states may share common neuronal features.

## Materials and Methods

### Animals

Cockroaches (*Periplaneta americana*) were raised in crowded conditions in plastic containers (50 X 50 X 70 cm) under a 12D:12L cycle at 26°C. Water and food (catfood) were provided *ad libitum*. Wasps (*Ampulex Compressa*) were reared in custom Perspex cages (40 X 50 X 50 cm) at 30°C with 40% humidity on a 12D:12L cycle. Wasps were provided 20% sucrose in water, honey and water *ad libitum*.

### Behavior

Adult male cockroaches with intact antennae were placed in an arena 5 hours after having being stung or un-stung. To induce quiescence, we used a protocol described in Watson and Ritzmann (1994). By positioning a plastic petri dish over the cockroach that touched both antennae (referred to as 'antennal-contact'), several attempts to induce quiescence were made. The duration of induced quiescence of both free-ranging and tethered cockroaches was measured using a stopwatch. Tactile stimuli to the last abdominal segments (3 seconds interval stimuli) with a no.1 artists brush were used to assess the level of responsiveness of the animal, and the stimulus was repeated until the cockroach either regained alertness and performed a stereotypic escape response or initiated spontaneous locomotion. For electrophysiological recordings, we used either stung cockroaches or cockroaches that entered quiescence in more than 80% of the trials.

### Electrophysiological Recordings

Cockroaches were cold-anesthetized and immobilized in modelling clay. An electromyogram (EMG) bipolar electrode, made of two 38μm formvar coated nichrome wires, was inserted manually into the coxal depressor muscle of the metathoracic leg (muscle 177d). This muscle is controlled by only two excitatory motoneurons, the slow and fast coxal depressor motoneurons (Ds and Df motoneuron, respectively) [10,25]. Therefore, the EMG spikes are one to one with motoneuron recruitment, thus providing a direct read-out of the motor output from the metathoracic ganglion. After the insertion of the electrode, the cockroach was released and, after at least 5 minutes of accommodation, electrical activity in the muscle was recorded. This protocol was used in awake, quiescent, or stung cockroaches. Quiescence was induced in the same manner as in the behavioral trials (with a half petri dish).

To test the involvement of the head ganglia and specifically the CX in the control of the Ds motoneuron activity, EMG recordings from the coxa were combined with focal procaine injection into the CX. Procaine is a reversible sodium voltage-dependent channel blocker which non-selectively prevents action potential in the site of injection when injected in the cockroach’s central nervous system [23]. When injected into the CX, procaine is known to induce a 'stung'-like or a 'quiescent'-like behavior [23]. In addition, procaine is known to effectively block neural activity near the injection site in the CX [26]. After the insertion of the EMG electrode into the immobilized cockroach as described above, a U-shaped pin was placed on the neck to reduce hemolymph flow to the head. A rectangular-shaped incision was then made on the head capsule to access the brain. A glass needle was used to inject 9nl of procaine (500 mg/ml, or saline as a control) directly inside the CX. The inert tracer Janus Green (2%) was dissolved in all solutions to allow *postmortem* verification of the injection site. After being injected with procaine or saline and having its head incision sealed with wax, the cockroach was released. Electrical activity in the depressor muscle was recorded immediately after injection and until the recovery from procaine was behaviorally apparent.

The EMG recording was amplified with a high-gain differential amplifier (AM systems model 1700), and acquired with a Micro 1401 (CED) data acquisition unit (DAQ), recorded as waveform data and saved directly to a computer. The onset of Stimulus was also recorded through the DAQ.

### Histology

At the end of the experiment, the head was severed and placed in formalin (Sigma) overnight. The brain was then excised from the head, embedded in agar (6% agar in saline, Sigma), and sliced with a vibratome (Leica VT 1000S). The location of the Janus Green tracer in the brain was identified using a light microscope. Only trials in which the CX was confirmed as the injection site were used for further analysis.

### Analysis and Statistics

Behavioral data regarding the response to tactile stimuli was analyzed by averaging the number of stimuli needed to induce an escape response for each of the three groups of cockroaches ("Awake", "Quiescence" and "Stung"). Only trials that resulted in an escape response were considered for the statistical analysis.

Waveform data from the electrophysiological recordings was analyzed offline using Spike2 software (CED) and then exported to Excel. Only spikes with amplitude of at least twice that of the background noise were considered. For all EMG recordings, the number of spikes per second (spike/s) was calculated and then averaged for each group of cockroaches. In addition, the same data was analyzed using an interval histogram analysis, at a 0.1 seconds Interval Length. This data was normalized by dividing the number of intervals in each bin by the total number of intervals for each cockroach and then averaged among cockroaches from the same group.

All statistical tests were performed using SigmaPlot 13.0 software. The statistical significance was determined by using t-test, one-way ANOVA, Kruskal-Wallis one-way ANOVA on Ranks or Friedman Repeated Measures ANOVA on Ranks. For 'All Pairwise Multiple Comparison' procedures, the test recommended by 'SigmaPlot' was used.

## Results and Discussion

### Behavior

Quiescence induction (S2 File) was attempted for 11 consecutive trials (at an interval of at least 10 seconds) in 41 free ranging and tethered cockroaches (pulled together for lack of statistical difference in the average values between both groups). An average success rate of 83.4% (±0.04 SEM) per cockroach revealed that antennal-contact induces quiescence reliably. The duration of such induced quiescence, if the cockroach was allowed to wake spontaneously, ranged from 5 to 610 seconds (Average±SEM = 102.8±20.8; n = 25).

Quiescent cockroaches remained motionless and did not engage in locomotory activities such as grooming or walking. The body was positioned parallel to the ground with a low posture, head down with the mouth parts and antennae motionless; the latter were resting limply on the surface. Such a posture has been described in other insects as well [27]. For instance, the bodies of resting bees sink down to the substrate, indicating a decrease in leg muscle tone [27].

The responsiveness to stimuli in awake, quiescent and stung cockroaches (Awake; n = 14, 42 trials; Quiescence; n = 20, 76 trials and Stung; n = 7, 29 trials) was measured by the number of stimuli required to induce an escape response. Awake cockroaches reacted with an escape response to the first stimulus in every trial (42 trials). Quiescent cockroaches generally reacted to repeated stimuli (54 trials); however some quiescent cockroaches escaped after the first stimulus (21 trials) or did not escape at all (1 trial). None of the stung cockroaches performed an escape response after the first stimulus and most of the stung cockroaches did not escape even after repetitive stimuli (26 trials), except in 3 trials when escape did occur. The average number of stimuli±SEM required to induce an escape response (only of the trials that resulted in escape) was found to be significantly different among the three groups (P<0.001, Awake = 1.0±SEM, Quiescence = 3.2±0.2 and Stung = 6.0±1.0).

The fact that quiescent cockroaches reacted to repetitive stimuli with an escape response shows that quiescent cockroaches have an elevated arousal threshold and also that the quiescent state is reversible with stimulation. Similarly to the quiescent cockroach, the stung cockroach also displayed a heightened threshold for responding to stimuli. However, in contrast to quiescence, the number of stimuli required to induce an escape was higher and in general, even repeated stimuli did not induce an escape response. In addition, the venom-induced lethargy is long-lasting and reverses only after 5 to 7 days [28]. That being said, stung cockroaches can be taken out of the venom-induced lethargic state if challenged with a stressful stimulus such as water immersion, which induces immediate walking, or if placed upside-down, which induces immediate righting [5,28]. But the similarity in posture in the quiescent and stung states raises the possibility that these two immobile states may share a common circuitry and that the stung state may represent an extreme version of the quiescent state.

Both the stung lethargic state and the quiescent state have some similarities to a naturally occurring rest state in cockroaches that is correlated to daytime [29]. This rest state, studied and described by Tobler and Neuner-Jehle, could be regarded as a sleep state since it fulfils all four behavioral criteria established to distinguish between various states of inactivity and sleep in invertebrates, including sleep rebound after sleep deprivation [30]. This is in accord with evidence of the existence of sleep states in other insect such as bees and flies [27,31]. The behavioral similarities between the stung state, quiescence. and sleep could be controlled by the same neuronal circuitries in the cockroach brain and the wasp might be tapping into such circuitries to induce a long lasting sleep-like state of its prey. Indeed, it is very possible that the evolution of the jewel wasp sting took advantage of a pre-existing quiescent state in its prey. While it is unlikely that the effect of the wasp sting confers a selection benefit on the cockroach, the quiescent state would be selected for because it increased survival by reducing the probability of detection by motion sensitive predators in the light. With this in mind, we investigated the neural correlates of quiescence and stung states and looked for similarities.

### Neuronal Correlates of Lethargy

**Fig 1:**
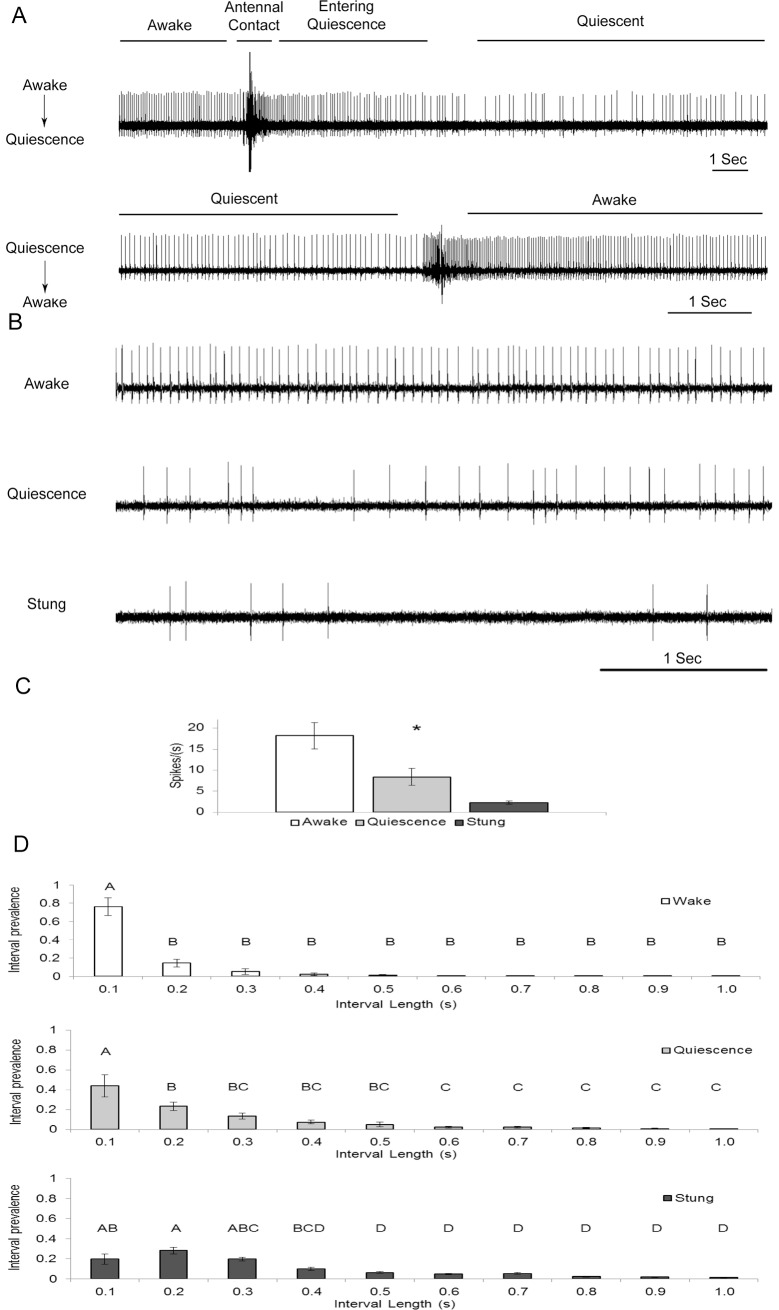
Activity in postural motoneuron (Ds) recorded as EMG Spikes from the coxal depressor muscle. (A) Transition between awake and quiescence. Top trace: Change in Ds activity before, during, and after the transition between awake and quiescent states in the same animal. The transition from awake state to quiescent state following antennal-contact induced quiescence is accompanied by a decrease in Ds firing rate. The large amplitude spike is an artifact occurring during antennal contact. Bottom trace: The transition from quiescent state to awake state in the same animal occurs spontaneously with a short burst followed by an increase in Ds firing rate. (B) Representative EMG recording traces of Ds ongoing activity in awake (top trace), quiescent (middle trace) and stung (bottom trace) immobilized cockroaches. (C) Each bar represents the average spikes/second±SEM (Awake = 18.2±3.2; Quiescence = 8.4±2.0 and Stung = 2.3±0.4). The average value for each group was significantly different from the other two groups (P<0.001): the Awake group displayed the highest muscle tone and the Stung group the lowest (n = 12 for each group). (D) Interval histograms of Ds spikes in awake (top histogram), quiescent (middle histogram) and stung (bottom histogram) cockroaches. Each bar represents the normalized number of spike intervals in each time bin (interval prevalence) ±SEM. Data points labeled with different letters are significantly different from each other (P<0.001 for 'A' label and P<0.05 for 'B-D' labels). (n = 12 for each group).

Using EMG recordings from the coxal depressor muscle of the metathoracic leg, the transition between awake and quiescent states of the cockroach was explored (Fig 1A). The transition from awake state to quiescent state that is induced by antennal-contact was accompanied by a gradual decrease in the Ds firing rate. We will refer to the firing rate of such postural motoneuron as muscle tone. The transition from quiescent state to awake state was spontaneous and the Ds firing rate was restored to generate more muscle tone. Using such recordings, we examined the change in muscle tone between the three groups of cockroaches under examination (n = 12 for each group). Fig 1B shows a representative example of muscle electrical activity caused by the recruitment of the Ds motoneuron firing while in a standing posture. Typically, an awake cockroach will display a continuous and spontaneous Ds firing at a fairly constant rate. Quiescent and stung cockroaches display a decreased and more variable firing rate of Ds. Quantification of these results revealed a significant difference in muscle tone between the three groups (P<0.001, Fig 1C). Awake cockroaches exhibited the highest muscle tone, followed by quiescent cockroaches with a lower muscle tone, and stung cockroaches with the lowest muscle tone (average spikes/second ±SEM: Awake = 18.2±3.2; Quiescence = 8.4±2.0 and Stung = 2.3±0.4). Postural motoneurons are known to decrease the firing rate during rest-states in both insects [27] and mammals [32].

Moreover, the relatively-constant spike intervals in awake cockroaches became more variable in quiescent and stung cockroaches (Fig 1B) suggesting that during venom or antennal induced resting state, Ds motoneuron does not receive tonic descending input from the head ganglia. To confirm this observation, we further evaluated the Ds firing rate with a spike interval histogram (Fig 1D). The interval histogram shows that, for awake and quiescent cockroaches (n = 12 for each group), the Ds firing intervals are fairly constant, ranging from 0 to 0.1 seconds. In contrast, in stung cockroaches (n = 12), the Ds firing intervals are spread over a wide range, with no preferable interval. In addition, the median values of the three groups were found to be significantly different (Friedman repeated measures ANOVA on Ranks, P = 0.002) which demonstrate the difference in the 'interval prevalence' distribution between the three groups.

**Fig 2:**
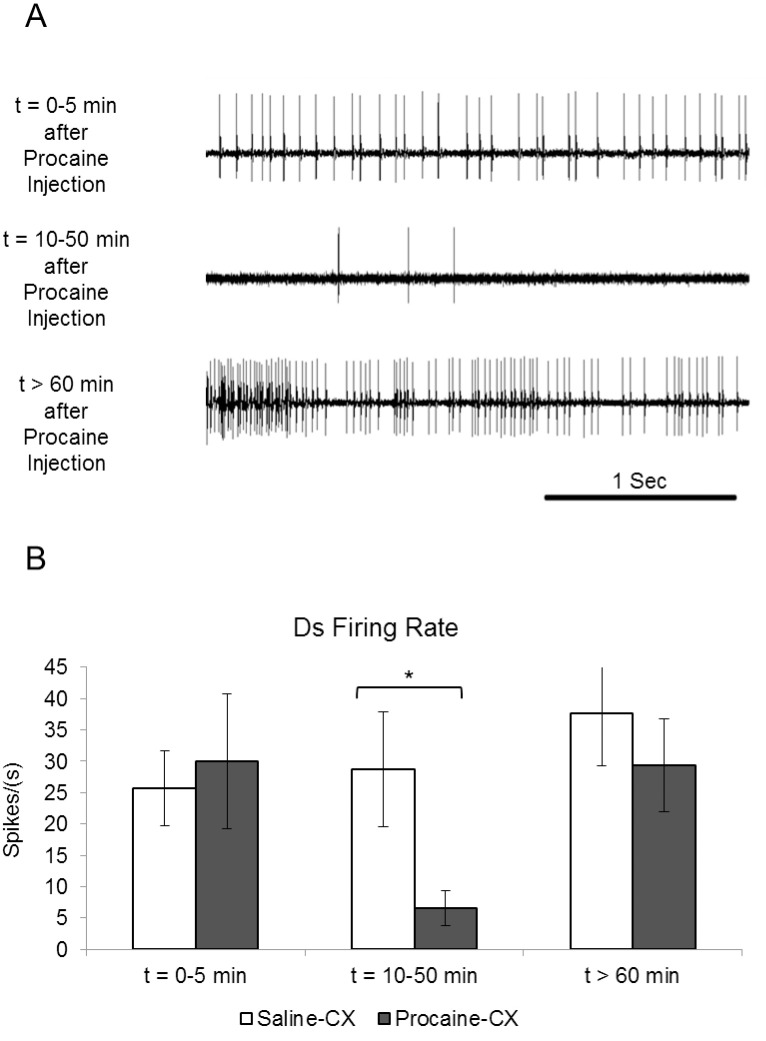
Activity in postural motoneuron (Ds) recorded as EMG Spikes from the coxal depressor muscle after procaine injection to the CX. (A) Representative EMG recording traces of Ds ongoing activity after procaine injection to the CX. (B) Each bar represents the average spikes/second ±SEM. The average values of the t = 10–50 min time point was significantly different for the two groups (P<0.05). (Procaine-CX; n = 6; Saline-CX; n = 6).

The Jewel Wasp stings cockroaches directly into their brain in order to control their ability to move. Such behavioral manipulation is achieved by the injection of venom inside and around the central complex (CX), a neuropil in the supra-esophageal ganglion (SupEG or “brain”). The CX is known to be involved in sensory integration and pre-motor processing [13] as well as in ongoing regulation of locomotion [3,17–23]. We have previously shown that focal injection of procaine or venom to the CX is sufficient to induce a pronounced decrease in the initiation of spontaneous walking [23]. The tonic firing of Ds during posture could be generated by endogenous properties of the motoneuron, by a local thoracic premotor circuitry, or by the descending tonic input from the head ganglia. To investigate the involvement of the head ganglia in the control of the Ds motoneuron firing pattern, we used focal injection of procaine, a reversible sodium voltage- dependent channel blocker (see Kaiser and Libersat 2015), directly to the CX. Fig 2A shows the Ds activity changes after injection of procaine to the CX. First, the baseline activity of Ds was sampled between time 0 and 5 minutes after the injection prior to the effect of procaine. Ds activity decreased approximately 10 minutes after injection and the activity seemed less tonic as recorded for a period of 40 minutes. After recovery from procaine, which occurs roughly 60 minutes after injection, the Ds activity increased and resumed tonicity. Quantification of these recordings is shown by comparing the Ds firing rate of procaine- or saline-injected cockroaches (Procaine-CX; n = 6; Saline-CX; n = 6) for three relevant time intervals for procaine activity [23]: (1) between 0 and 5 minutes after injection ('t = 0–5 min', prior to the effect of procaine); (2) between 10 and 50 minutes after injection (t = 10–50 min, when the effect of procaine is maximal); and (3) more than 60 minutes after injection (t>60 min, when the effect of procaine has worn off) (Fig 2B). A significant decrease (P<0.05) in the Ds firing rate was found only in the time interval t = 10–50 min.

Regarding the fast motoneuron, Schaefer and Ritzmann (2001) have examined Df recruitment in decapitated cockroaches [33]. They showed that there is a loss or reduction in fast motor neuron activity. The loss of fast motor neuron activity was also observed in a reduced preparation in which descending neural signals were reversibly blocked via an isotonic sucrose solution. We show here (Fig 1A) that Df never fires if no escape or walking is initiated by a tactile stimulus in the quiescent state, thus confirming previous observations made by Fouad et al, (1996) [12]. Regarding the CX, Guo and Ritzmann (2013) have shown that most CX units show an increase in their firing rate prior to the initiation of locomotion, indicating that the CX promotes walking. In addition, monoamines have been found to have an impact on the CX thereby affecting various aspects of sleep regulation in drosophila [34,35]. Finally, injection of the monoamine octopamine in the CX partially restores walking in hypokinetic cockroaches stung by the wasp [36]. Procaine and wasp venom are both known to decrease neuronal activity [23,26] and they both have a similar, though not identical, effect on Ds when injected in and around the CX (Fig 2). Hence, all evidence suggests that the behavioral change displayed by quiescent and stung cockroaches that is accompanied by a decrease in Ds motoneuron activity might be mediated by the head ganglia pre-motor circuits; specifically the CX.

To summarize, both the quiescent and the stung states differ significantly from the awake state with regard to: 1) an increased threshold for initiation of walking and 2) decreased postural Ds motoneuron activity. These changes might be mediated by the head ganglia pre-motor circuits since injection of Procaine to the CX decreases Ds motoneuron activity in a manner similar to the wasp venom injection in the CX. These results suggest that the venom-induced lethargic state in cockroaches might represent an extreme version of the quiescent state and that both states might be controlled by the head ganglia. If true, this implies that, in the course of the coevolved arms race between a parasite and its host, the parasite has found ways to tap into an existing neuronal circuit which is part of the quiescence-regulating network for its own benefit.

## Supporting Information

S1 File The escape response of stung and un-stung cockroaches. The video displays the normal escape response of a cockroach after a tactile stimulus to the abdomen with a brush. Next, the altered escape response of a stung cockroach (marked with a white dot on back) is shown.
(WMV)

S2 File Antennal induced quiescence. An adult male cockroach is placed in an arena and a plastic petri dish is positioned over the cockroach and touching both antennae. The antennal contact induces quiescence in the cockroach, which exhibits a change in posture and lack of antennal and mouth-parts movement. Repetitive tactile stimuli with a brush does not evoke an escape response and only after waking, marked by the antennal movement, does locomotion occur.
(WMV)
